# Microbiome interplay: plants alter microbial abundance and diversity within the built environment

**DOI:** 10.3389/fmicb.2015.00887

**Published:** 2015-08-28

**Authors:** Alexander Mahnert, Christine Moissl-Eichinger, Gabriele Berg

**Affiliations:** ^1^Institute of Environmental Biotechnology, Graz University of TechnologyGraz, Austria; ^2^Interactive Microbiome Research, Section of Infectious Diseases and Tropical Medicine, Department of Internal Medicine, Medical University GrazGraz, Austria; ^3^BioTechMed Interuniversity Cooperation CentreGraz, Austria

**Keywords:** interplay of microbiomes, indoor plants, built environment, 16S gene and ITS region amplicons, *Chlorophytum comosum*, qPCR, LEfSe analysis, network analysis

## Abstract

The built indoor microbiome has importance for human health. Residents leave their microbial fingerprint but nothing is known about the transfer from plants. Our hypothesis that indoor plants contribute substantially to the microbial abundance and diversity in the built environment was experimentally confirmed as proof of principle by analyzing the microbiome of the spider plant *Chlorophytum comosum* in relation to their surroundings. The abundance of Archaea, Bacteria, and Eukaryota (fungi) increased on surrounding floor and wall surfaces within 6 months of plant isolation in a cleaned indoor environment, whereas the microbial abundance on plant leaves and indoor air remained stable. We observed a microbiome shift: the bacterial diversity on surfaces increased significantly but fungal diversity decreased. The majority of cells were intact at the time of samplings and thus most probably alive including diverse Archaea as yet unknown phyllosphere inhabitants. LEfSe and network analysis showed that most microbes were dispersed from plant leaves to the surrounding surfaces. This led to an increase of specific taxa including spore-forming fungi with potential allergic potential but also beneficial plant-associated bacteria, e.g., *Paenibacillus*. This study demonstrates for the first time that plants can alter the microbiome of a built environment, which supports the significance of plants and provides insights into the complex interplay of plants, microbiomes and human beings.

## Introduction

In recent years, deeper insight into the microbial diversity associated with plants and humans was gained using novel omics approaches; both are now recognized as meta-organisms: a functional unit of eukaryotic cells and microorganisms (Berg et al., 2014a). In contrast, the connection between microbiomes as well as the mutual exchange between them is less understood (Blaser et al., 2013). Although we live in a highly interconnected world, until the present date only a few examples of synergistic microbiomes have been discovered, which have shown that there are important relationships between single microbiomes (Berg, 2015). The rhizosphere is a well-investigated example that presents the root-soil interface influenced by the plant via root exudates as well as by the soil microbiome (Philippot et al., 2013). For instance, the rhizosphere mainly selects bacteria from soil but also contains indigenous plant-associated bacteria, e.g., bacteria derived from seeds (Fürnkranz et al., 2012). While the rhizosphere is an example of particular importance for plant health, human health is for instance strongly dependent on the gut microbiome. David et al. (2014) recently provided evidence for the food-gut connection by analyzing the survival and metabolic activity of foodborne microbes from a plant-based diet after transit through the digestive system. Whereas this study highlighted the influence of plant-associated microbiota on the human gut microbiome, nothing is known about the impact of the phyllosphere-associated microbiota (Vorholt, 2012) on microbial abundance and diversity in the built environment. Indoor environments are considered to have big impact on human health (Reponen et al., 2012), since people in developed countries spend most of their lifetime indoors.

Built environments are not only habitats for humans; they also can be considered as biotopes for diverse microbes, whereas their abundance was mainly attributed to the presence of humans and their pets (Hanski et al., 2012; Kelley and Gilbert, 2013; Lax et al., 2014). Until now the significance of plants for humans and the built environment was mainly seen in psychological effects like mood and comfort behavior or VOCs (volatile organic compounds) as well as removal and improvement of indoor air (Sriprapat et al., 2014), but has never been linked to plant-associated microorganisms. However, is it possible that indoor plants function like humans as important or even valuable microbial dispersal sources? Our hypothesis that indoor plants contribute substantially to the microbial abundance and diversity in the built environment was already published as opinion (Berg et al., 2014b). Our hypothesis was based, amongst others, on the observation that hospital rooms that were window ventilated, contain plant-associated bacteria with potential beneficial traits for the eukaryotic hosts (Oberauner et al., 2013).

The objective of this study was to confirm our hypothesis by performing an experiment as a proof of principle, where we tracked the *Chlorophytum comosum* microbiome toward its surroundings inside an enclosed indoor environment. The spider plant *C. comosum* (Thunb.) Jacques is a monocotyledonous plant (Family *Asparagaceae*) and one of the most common indoor plants world-wide. Spider plants have been shown to have a positive impact on indoor air quality by efficiently reducing air pollution such as formaldehyde, toluene, and ethylbenzene (Sriprapat et al., 2014). Our results indicate that the plant associated microbiome spreads into the environment and might thus allow an interaction of human and plant associated microbiomes inside the built environment, which could be much more important than it had ever been assumed before.

## Materials and methods

### Experimental design

The common indoor plant *C. comosum* was kept isolated in a pre-cleaned chamber (2.27 m^3^) for almost half a year. During the period of isolation the microclimate was monitored with respect to temperature and relative humidity. Samples for molecular analysis covered the indoor air (1.17 m^3^), plant leaves (0.16 m^2^), and surrounding surfaces (glass and press board walls and, floor tiles; 0.811 m^2^) within the chamber. The plant had been part of an office inventory before it was transferred to the clean chamber. The surfaces of the chamber and all other abiotic surfaces (e.g., plant pot) were cleaned in several steps to remove microbial and DNA remnants, to be able to identify the plant's contribution to the indoor microbiome after the incubation period. First, surfaces were cleaned with water and detergents (all-purpose cleaner, Denkmit, dm-drogerie markt GmbH + Co. KG, Karlsruhe, Germany), followed by cleaning with 70% (w/v) ethanol (Carl Roth GmbH & Co KG, Karlsruhe, Germany) and Bacillol® plus (Bode Chemie GmbH, Hamburg, Germany) to remove most microbes. Chlorine bleach (DNA away, Molecular Bio Products, Inc. San Diego, CA, USA) and UV light (254 and 366 nm, Kurt Migge GmbH, Heidelberg, Germany) was used to fragment and remove remaining DNA in the chamber. The plant was placed on a pedestal in the chamber and watered once a week. Natural tab water was selected to sustain hydration of the plant. This procedure was preferred over a supply with sterilized water and soil to be more comparable with common house plants. Beside the sampling events, watering of the plant was the only period of time where the chamber was opened for some seconds and potentially susceptible to the surrounding laboratory environment. This potential input from the adjacent built environment was covered by a control (see below). Supply with light was guaranteed by natural sun light through glass windows and supported by an artificial light source according to the day/night cycle. Samples were taken in the following order: First samples from the surfaces of the cleaned chamber were received from floor and wall surfaces (surface_t0). Then samples from plant leaves were sampled before the plant was transferred to the cleaned chamber to avoid any artificial spreading of microbes due to the sampling procedure itself (plant_t0). Sampling the air (air_t0) of the chamber with the plant inside finalized all sampling steps for time point and sample group t0. Plant growth could be observed during the time of incubation. The plant was positioned on a pedestal with a reasonable distance (radius of ~80 cm) to the surrounding wall and floor surfaces (distance of ~36 cm to wall and floors, ~100 cm to the ceiling). The plant had an initial volume of about 225 cm^3^ and doubled its volume during the incubation period. Incubation was stopped, when the first plant leave made direct contact with the surrounding indoor surface (contact to the floor surface due to leave growth of ~36 cm), to avoid direct transfer of phyllosphere associated microbes onto surfaces. However, throughout the incubation period, seed and flower particles were shed onto the floor surface. After the incubation period samples were taken in the following order to obtain sample group t1: First the air was sampled inside the chamber (air_t1). Then the plant was carefully removed from the chamber and the plant leaves were sampled (plant_t1). Finally surfaces of the empty chamber were sampled (surface_t1).

### Sampling procedure

Indoor air samples were obtained using the SKC BioSampler® (SKC Inc., PA, USA). All parts of the air sampler were autoclaved at 121°C for 30 min to achieve sterility and treated with dry-heat at 170°C for 24 h to degrade DNA (Probst et al., 2013). Four air sampling replications were processed in a serial manner at a flow rate of 13 l/min to allow an entire room volume to pass through the impinger (sampling of particles from the air into PCR-grade water, Sigma-Aldrich Chemie GmbH, Stiegheim, Germany, or Carl Roth GmbH & Co KG, Karlsruhe, Germany) in about 20 min. For one replica the procedure was repeated three times (within an hour) and resulting samples (10 ml each) were pooled (30 ml total volume). For sampling plant leaves and surrounding chamber surfaces in four replications, sterile (autoclaved) and DNA-free (dry heat treatment) Alpha Wipes® (TX1009, VWR International GmbH, Vienna, Austria) were used. Alpha Wipes® were extracted in 100 ml PCR-grade water, vortexed and sonicated at 40 kHz for 2 min. Sample extracts of air, plant leave and surface samples were concentrated 100-fold to 1 ml using Amicon Ultra-15 centrifugal filter tubes (Ultracel-50K, Merck Millipore KGaA, Darmstadt, Germany). Negative controls, field blanks, sequencing controls for prokaryotes and eukaryotes and additional PMA treatment of a sample subset were processed in parallel with all samples. This procedure allowed a quality control for the sample equipment, used reagents, background signals of the indoor environment and to which extent sequences were obtained from actual intact microbial cells. Results presented in this study are based on only those samples, which passed these rigorous quality controls through PCR-testing of respective samples and controls.

### PMA treatment and DNA extraction

PMA (propidium monoazide, GenIUL, S.L., Terrassa, Spain) treatment and DNA extraction of samples was applied as optimized and reported before (Moissl-Eichinger et al., 2015). PMA helps to determine the proportion of dead cells and free DNA in a sample, by masking free and non-membrane encased DNA in downstream processes such as PCR. Hence, after observing an over-proportional amount of intact cells compared to other enclosed indoor environments (Moissl-Eichinger et al., 2015) this procedure was not applied to all samples and represented an additional control for possible DNA contaminants and drawn conclusions of this study in general. Afterwards cells were mechanically lyzed in Lyzing Matrix E tubes filled with glass beads (MP Biomedicals, Heidelberg, Germany) on a FastPrep®-24 Instrument (MP Biomedicals, Illkirch, France) at 6.5 m/s for 2x 30 s. DNA was extracted according to the XS buffer method applicable for low biomass environments (Moissl-Eichinger, 2011).

### Quantitative PCR (qPCR)

For determining microbial abundance, qPCRs with bacterial (515f—927r; 10 μM each); fungal (ITS1—ITS2; 10 μM each); and archaeal (344aF—517uR; 5 μM each) directed primers were conducted (see Supplementary Table S1 for sequence of primers). The qPCR reaction mix for bacteria and fungi (7.04 μl) contained 5 μl QuantiTect SYBR® Green PCR kit (QIAGEN GmbH, Hilden, Germany), 0.2 μl BSA, 0.12 μl forward and reverse primers, 0.8 μl PCR grade water and 0.8 μl of the extracted genomic DNA as a template. For archaea targeted qPCR, the reaction mix (10 μl) comprised 3 μl PCR grade water, 5 μl QuantiTect SYBR® Green PCR kit (QIAGEN GmbH, Hilden, Germany), 0.5 μl forward and reverse primers (5 μM each), and 1 μl template DNA. A modified reaction mix (7 μl) was used for e.g., plant samples with observed amplification inhibitions, which might arose from plant associated inhibitory substances. 1.06 μl PCR grade water, 3.5 μl KAPA Plant PCR buffer (KAPA3G Plant PCR Kit, Peqlab, VWR International GmbH, Erlangen, Germany), 0.42 μl forward and reverse primers, 0.056 μl of KAPA3G Plant DNA-polymerase (2.5 u/μl), 0.78 μl of SYBR® Green (4x concentrate, Invitrogen™, Eugene, OR, USA), and 0.8 μl extracted DNA template.

Amplification of DNA templates and quantification of fluorescence was achieved on a Rotor-Gene™ 6000 real- time rotary analyzer (Corbett Research, Sydney, Australia) via the following PCR programs. Bacteria: 20 s at 95°C, 15 s at 54°C and 30 s at 72°C for 40 cycles followed by a melt curve from 72 to 95°C. Fungi: 40 cycles of 30 s at 94°C, 35 s at 58°C, 40 s at 72°C was used, and concluded with a melt curve. For archaea, 40 cycles of 15 s at 94°C, 30 s at 60°C, 30 s at 72°C was used followed by a melt curve. Ten individual qPCR runs with a mean reaction efficiency of 90% and R^2^ values of standard curves of 0.94 were performed separately and measured in triplicate. Occasional gene copy numbers found in negative controls were subtracted from their respective samples.

### Preparation of 16s rRNA gene and ITS region amplicons

Amplicons were prepared with two different barcoded primer combinations: 520f—802r specific for bacteria and ITS1f—ITS2rP regions specific for fungi (see Supplementary Table S1 for sequence of primers). Due to scattered PCR inhibitions (e.g., plant samples) for some samples Taq&Go™ Mastermix (MP Biomedicals, Heidelberg, Germany) was substituted with KAPA3G Plant PCR Kit and nested PCR procedures were applied to add barcoded primers. 1 μl template DNA was amplified on a Whatman Biometra® Tpersonal and Tgradient thermocycler (Biometra GmbH, Göttingen, Germany) and a TECHNE TC-PLUS gradient thermocycler (Bibby Scientific Ltd, Stone, UK) with the following cycling conditions: initial denaturation 95°C 5 min, denaturation 95°C 50 s, annealing 60°C 30 s (62°C 35 s for ITS regions), extension 72°C 60 s (40 s for ITS1-2). Four individual PCR reactions à 30 μl (6 μl Taq&Go™ polymerase, 18 μl PCR grade water, 1.5 μl forward and reverse primer (5 μM), 1 μl template DNA) or 50 μl (17.6 μl PCR grade water, 25 μl KAPA3G Plant PCR buffer, 0.4 μl KAPA3G Plant DNA-polymerase (2.5 u/μl), 3 μl forward and reverse primer (5 μM) and 1 μl template DNA) were pooled and transferred on a DNA free 96 well plate. The following pre-sequencing preparations were conducted by Eurofins Genomics GmbH, Ebersberg, Germany. According to HT DNA-QC (Agilent Technologies Sales & Services GmbH & Co.KG, Waldbronn, Germany) samples were pooled in equimolar concentrations in 2 pools (Pool_Bac520_Gelex and the Pool_Fungi_Gelex with 24 barcoded samples each). Library pools were provided with 2 different adaptor versions to increase complexity of samples. After quality control libraries were purified via gel extraction, quantified, and mixed. Sequencing was achieved on an Illumina MiSeq instrument with chemistry version 3 (2 × 300 bp). Reads were filtered and sorted according to inline barcodes and individual sequencing tags. Raw reads were deposited in the European Nucleotide Archive (www.ebi.ac.uk) under project PRJEB8807 (ERP009846).

### Bioinformatics and statistics

Filtered and sorted reads were additionally length- (200–400 bp) and quality filtered (phred q20) in QIIME (Caporaso et al., 2010). Chimeric sequences were identified and removed with usearch (Edgar, 2010) using either Greengenes gg_13_8 for 16S rRNA gene reads or UNITE ver6_99_s_04.07.2014 for ITS region amplicons as a reference. OTUs (operational taxonomic units) were picked according to the open reference given above and any sequence not present in the respective reference was clustered denovo with usearch (according to 16S analysis tutorial in QIIME) and uclust for ITS reads (according to the Fungal ITS analysis tutorial in QIIME). After OTU picking, representative sequence alignment, taxonomy assignment, and tree construction, an OTU table with all metadata was generated. The rarefied OTU tables (520f—802r 4062 sequences; ITS1f—ITS2rP: 6839 sequences) served as the main input for following alpha and beta-diversity analysis. Core OTUs at 100% were calculated for each category (air_t0, air_t1, plant_t0, plant_t1, surface_t0, surface_t1) and served as input for network analysis (see Moissl-Eichinger et al., 2015 for more details) and LEfSe analysis (Segata et al., 2011) calculated with Galaxy modules provided by the Huttenhower lab. Adonis, ANOSIM, MRPP and mantel tests were calculated in QIIME (using the vegan package in R) with 999 permutations (R Core Team, 2014). One and Two Way ANOVA and *t*-tests were calculated in R (R Core Team, 2014) and MS Excel.

## Results

### Abiotic parameters

Abiotic parameters (temperature, moisture) were constantly monitored to assess their impact on the microbial dispersal. The average temperature was 21.9 ± 3.2°C and reflects common conditions inside European buildings. A decrease of 13.4°C from 30.4°C (maximum temperature) in August to 17°C (minimum temperature) in December was observed (Supplementary Figure S1). Similarly, the average relative humidity showed a decrease from 66.7% (maximum) at the beginning of September to 18.7% (minimum) at the end of November with an average of 49.2 ± 9%. The day/night cycle resulted in a daily in/decrease of the average temperature from 0.3 ± 0.1 – 0.7 ± 0.6 °C (minimum 0.1°C in December to a maximum of 1.4°C in November) and 1.8 ± 1.1 – 3.4 ± 1.4% (minimum 0.1% in December to a maximum of 7.1% in September).

### The plant increased the microbial abundance in its environment

**Figure 1:**
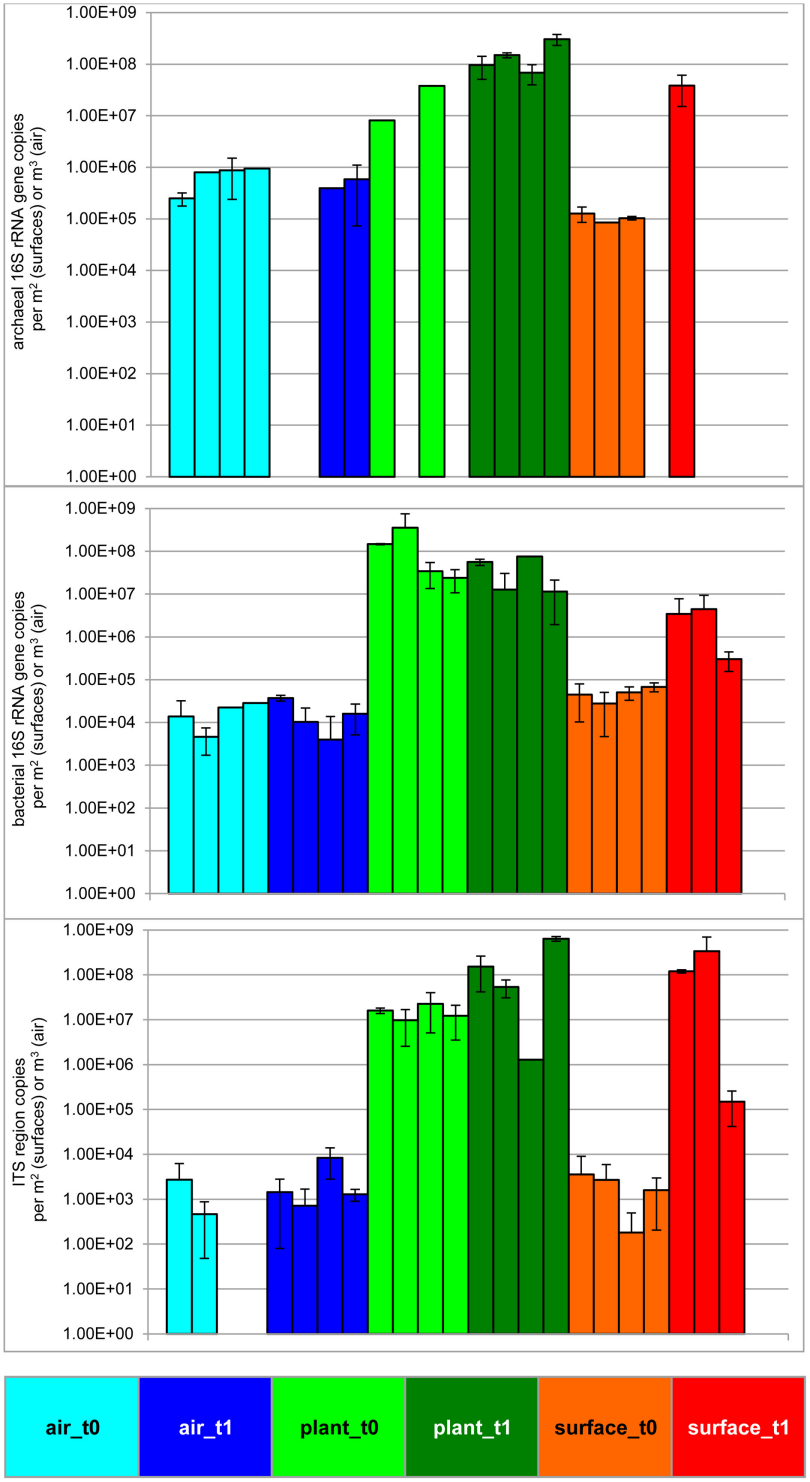
**Microbial abundance before and after plant isolation in a cleaned sealed chamber**. Blue bars represent air samples, green bars—samples from plant leaves and red bars represent samples obtained from surrounding wall and floor surfaces. Brighter colors indicate the first time point (prior to isolation, t0), darker colors indicate the second time point (after plant isolation, t1) respectively. Upper panel shows results from archaeal directed 16S rRNA gene primers, middle panel gives values from bacterial 16S rRNA gene copies and lower panel presents results obtained by primers targeting the ITS region of fungi. Samples from surfaces are calculated per 1 m^2^ and samples from the air are given per 1 m^3^.

The statistically significant increase (*t*-test *P* = 0.05) of microbial abundance on surfaces (walls and floor) was visible after 6 months of plant isolation in an indoor environment (Figure 1 and Table 1). The extent of increase was variable: the highest increase was determined for fungi (ITS region copies; up to 5 logs). For 16S rRNA gene copy numbers of Bacteria and Archaea an increase of up to 2 logs was detected. In contrast to the surrounding surfaces, the microbial abundance in the air and on plant leaves remained constant. An analysis of variance (ANOVA) showed significant variation of samples obtained from the indoor air, plant leaves, and surfaces for Archaea (*P* = 7.9^*^10^−5^), Bacteria (*P* = 1.5^*^10^−3^) and fungi (*P* = 7.9^*^10^−4^).

**Table 1:** **Summary of changes in abundance and diversity of an isolated indoor plant (***Chlorophytum comosum***) according to sampled indoor spaces (air, plant leaves, floor, and wall surfaces) and time of isolation (before plant incubation—t0, after plant incubation—t1)**.

	**Indoor space**					
	**air_t0**	**air_t1**	**plant_t0**	**plant_t1**	**surface_t0**	**surface_t1**
Mean archaeal qPCR copy numbers per m^2^ or m^3^	7.27E+05	5.39E+05	2.32E+07	1.55E+08	1.09E+05	3.83E+07
Mean bacterial qPCR copy numbers per m^2^ or m^3^	1.32E+04	1.98E+04	1.40E+06	3.37E+07	4.78E+04	2.38E+06
Mean fungal qPCR copy numbers per m^2^ or m^3^	1.91E+03	3.41E+03	1 50E+07	2.17E+08	1.09E+05	1.31E+08
Mean Shannon-Wiener index (H') bacteria	5.39	5.31	6.15	6.94	4.82	6.90
Mean Shannon-Wiener index (H') fungi	3.87	6.53	7.20	6.28	7.14	4.98
LEfSe analysis summary—taxonomic assignment of OTUs and respective LDA score (log 10)		*Acidovorax* (4.1), *Methylobacterium* (5.3)	*Cyanobacteria* (4.8)	*Saprospirae* (4.2), *Saprospirales* (4.1), *Alteromonadaceae* (4.3), *Alteromonadales* (4.3), *Bacteroidetes* (5.0), *Cellvibrio* (4.3), *Chlamydiae* (4.2), *Chlamydiales* (4.3), *Chlamydiia* (4.3), *Clostridium intestinale* (4.0), *Cytophagaceae* (4.9), *Cytophagales* (4.9), *Cytophagia* (4.9), *Devosia* (4.2), *Dyadobacter* (4.5), *Flavobacteriales* (4.0), *Flavobacteriia* (3.9), *Gemmatales* (4.3), *Gemmatimonadetes* (3.9), *Gemmatimonadetes* (3.9), *Legionellaceae* (3.9), *Legionellales* (3.9), *Luteimonas* (4.1), *Methylocystaceae* (4.2), N1423WL (3.9), *Parachlamydiaceae* (4.3), *Planctomycetes* (4.4), *Planctomycetia* (4.3), *Rhizobium* (4.1), *Sphingopyxis* (3.9), *Xanthomonadales* (4.5)	*Bradyrhizobium* (4.1), TM7 (4.1), TM7_3 (4.0)	*Acidobacteria* (4.8), *Acidobacteria_*6 (4.9), *Bacilli* (4.8), *Chloroflexi* (4.0), *Paenibacillaceae* (4.2), *Rhodospirillales* (4.3), *Heterobasidion* (5.9), *Bondarzewiaceae* (5.9)

### The plant increased the microbial diversity in its environment

Microbial diversity was assessed by analyzing amplicon pools, which comprised 1,351,533 (bacteria) and 1,903,469 (fungi) quality sequences with 56,298 (bacteria) and 185,252 (fungi) picked OTUs at a 97% similarity level (Supplementary Tables S2–S4). The diversity changed during the time of incubation (Table 1). Whereas the mean bacterial diversity (calculated with the Shannon-Wiener index: H') remained almost stable on plant leaves and in the air (H' 6.15–6.94 and H' 5.39–5.31), bacterial diversity increased significantly on surrounding wall and floor surfaces (H' 4.82–6.9, *t*-test *P* = 7.8^*^10^−33^). On the contrary fungal diversity decreased significantly on surfaces (H' 7.14–4.98, *t*-test *P* = 1.2^*^10^−17^) and in the air (H' 3.87–6.53, *t*-test *P* = 8.62^*^10^−19^), but remained again almost stable on plant leaves (H' 7.2–6.28).

**Figure 2:**
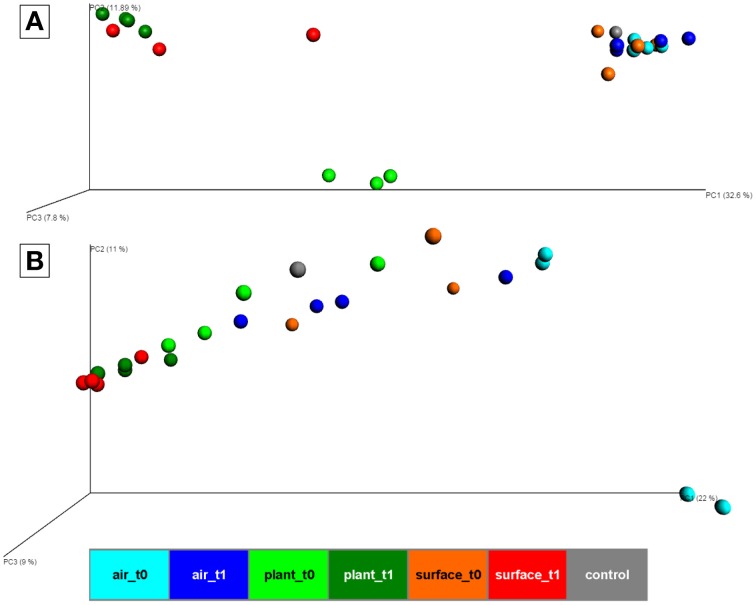
**PCoA plot with scaled coordinates by percent explained based on Bray-Curtis distances of rarefied OTU tables (4062 sequences for bacteria and 6839 sequences for fungi)**. **(A)** shows results of the bacterial 16S rRNA gene amplicons. **(B)** shows results of the fungal ITS amplicons. Spheres are colored according to the indoor space and the time points as highlighted in Figure 1. The control in gray was a sample from the lab environment outside the chamber after the isolation period.

At the beta-diversity level, three distinct clusters appeared in a principal coordinate analysis based on Bray-Curtis distances of bacteria (Figure 2A). The first cluster was composed of samples from the air and the surrounding chamber surfaces prior to the plant isolation and the control. This cluster showed reasonable distance along PC1 axis (with a high variation of 32.6% explained) to the second cluster formed by plant leave samples prior to the isolation and the third cluster comprising samples from plant leave samples and surrounding surfaces after the isolation period. The ordination for fungi (Figure 2B) showed no distinct clusters of different sample groups, but similar changes in diversity along the PC1 axis (with a high variation of 22% explained). One of the most important findings was that indoor surfaces showed higher similarity to plant leaves after the isolation period. For bacteria, the calculated mean Bray-Curtis distances changed significantly (*t*-test *P* = 1.7^*^10^−10^) from 0.9 (surface_t0 vs. plant_t0) to 0.67 (surface_t1 vs. plant_t1) with a mean distance of all samples at 0.63. Likewise for fungi the calculated mean Bray-Curtis distances changed significantly (*t*-test *P* = 2.6^*^10^−10^) from 0.75 (surface_t0 vs. plant_t0) to 0.37 (surface_t1 vs. plant_t1) with a mean distance of all samples at 0.59. However, a similar trend for samples from the indoor air although less significant (*t*-test *P* = 0.001, due to a high sample dispersal) could only be perceived for the fungal communities 0.86 (air_t0 vs. surface_t0) to 0.73 (air_t1 vs. surface_t1). An adonis test (55% variation explained for bacteria and 44% for fungi) and an analysis of similarities (ANOSIM, R-statistic = 0.68 for bacteria and 0.3 for fungi) showed significant (*P* = 0.001) grouping of samples by their categories at an alpha of 0.05 with a stronger grouping per individual for bacteria. A Monte-carlo permutation based analysis (MRPP) between samples obtained from air, plant leaves, and wall and floor surfaces before and after plant isolation, resulted in a delta of 0.001 and a chance corrected within-group agreement of 0.2038 for bacteria and 0.1628 for fungi. Hence, the MRPP revealed significant differences between the overall sampled communities.

### LEfSe analysis revealed plants as a new source for the microbiome within the built environment

**Figure 3:**
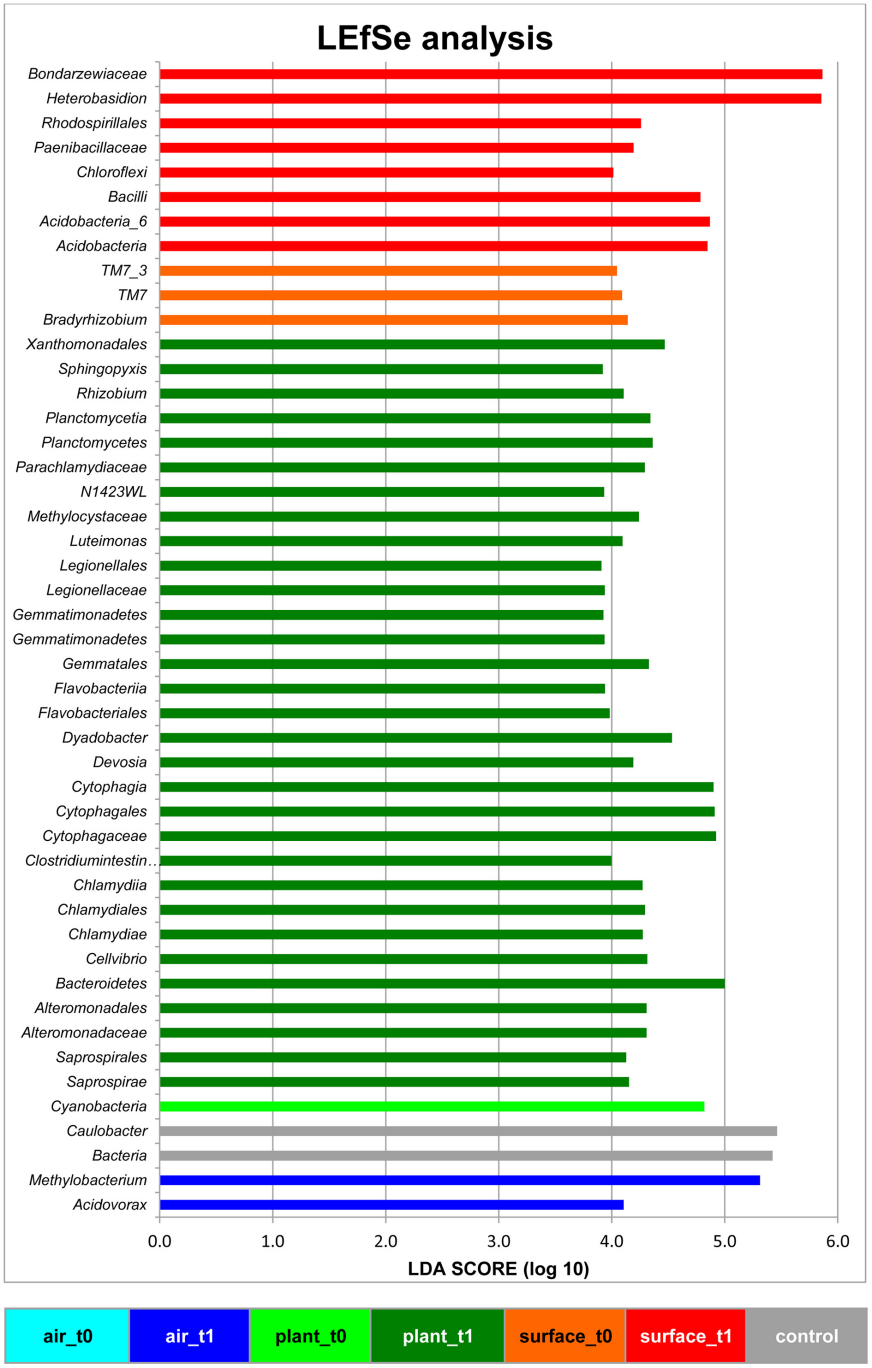
**Linear discriminant analysis Effect Size (LEfSe) of bacterial and fungal OTUs, which most likely explain differences between sampled indoor classes (indoor air, plant leaves, floor and wall surfaces prior and after plant incubation)**. Results were colored and grouped according to indoor classes as in Figure 1. The control in gray was a sample from the lab environment outside the chamber after the isolation period.

**Figure 4:**
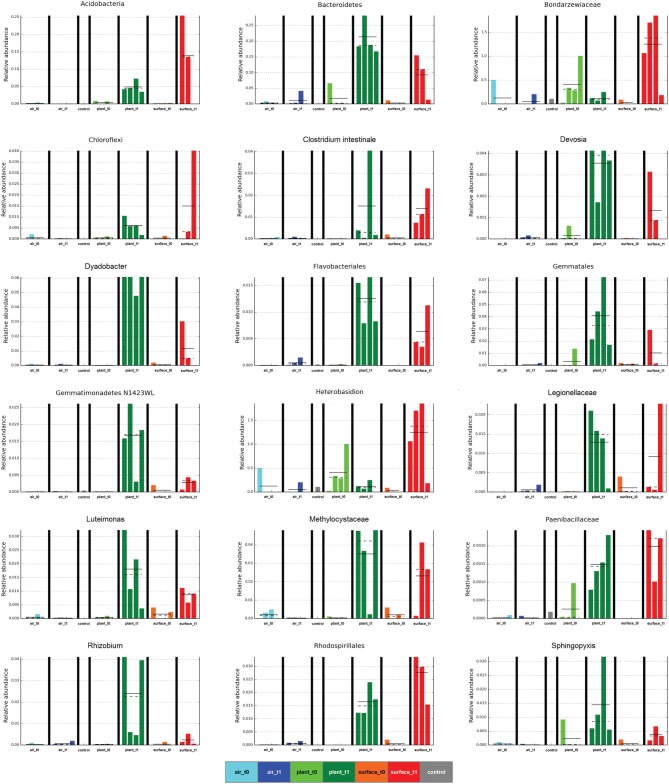
**Selected abundance histograms of features (sampled indoor spaces—indoor air, plant leaves, floor and wall surfaces prior and after plant incubation) detected by LEfSe as biomarkers**. Sample groups are colored according to Figure 1. The control in gray was a sample from the lab environment outside the chamber after the isolation period.

The linear discriminant analysis of the effect size [LEfSe; (Segata et al., 2011)] of bacterial and fungal core OTUs revealed features that most likely explained differences between sampled indoor classes. According to this analysis 47 OTUs could be identified to be responsible for discriminating between the different sampled spaces and microbiomes (Figure 3). Hence, amongst other OTUs from lower taxonomic levels, *Acidovorax, Methylobacterium* (for air_t1 samples); *Caulobacter* (for control samples); *Cellvibrio, Clostridium intestinale, Devosia, Dyadobacter, Luteimonas, Rhizobium, Sphingopyxis* (for plant_t1 samples); *Bradyrhizobium* (for surface_t0 samples); and *Heterobasidion* (for surface_t1 samples) were significantly responsible to explain differences of their respective indoor space. For a deeper insight some of these OTUs are shown as abundance histograms in relation to the sampled indoor environment (Figure 4). This analysis showed that mainly OTUs from plant samples and surrounding floor and wall samples were significantly responsible for discriminating the different categories of indoor environments and revealed that the plant serves as a source of microbes within the built environment.

The distribution of core OTUs according to their sampled indoor spaces substantiated results obtained by the LEfSe analysis and was visualized as a core OTU network for bacteria (Supplementary Figure S2) and fungi (Supplementary Figure S3). A detailed analysis of these distribution patterns showed that most core OTUs were shared between samples from time point t1. The surrounding floor and wall surfaces were the only category where an increase from 14.1% (bacterial OTUs) and 13.5% (fungal OTUs) before plant incubation (surface_t0) to 19.8% (bacterial OTUs) and 23.1% (fungal OTUs) after plant incubation (surface_t1) could be determined. On the contrary OTUs detected in control samples were shared to the lowest proportion (0.8% bacteria and 6.6% fungi).

**Figure 5:**
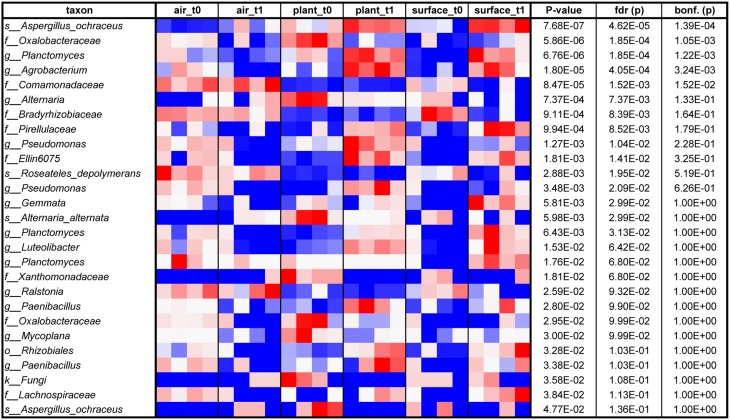
**Heatmap from blue (low) via white to red (high) of those taxa, which relatively increased on different indoor spaces (indoor air, plant leaves or wall and floor surfaces) sorted according to ***P***-value (at an alpha of 0.05 determined by an ANOVA; in addition false discovery rate (fdr) and Bonferroni corrected *P*-values (bonf.) are shown as well)**.

The air was dominated (>10,000 sequences) by sequences assigned to *Deinococcus, Bosea genosp., Delftia, Caulobacter, Methylobacterium, Volutella, Schizophyllum commune, Trametes versicolor*, and *Aspergillus ochraceus*. The same fungal genera (the last three named genera) and species could be found to high proportions on plant leaves together with the bacterial genera *Paenibacillus, Enhydrobacter*, and *Pseudomonas*. The surfaces showed a complex mixture of these genera and species. From these taxa especially *Methylobacterium* is a common resident of the plant phyllosphere, whereas *Caulobacter* for instance is mainly associated to aquatic environments but also with phosphate-solubilizing abilities and *Delftia* is an example of a well-known genus that colonizes abiotic and biotic surfaces such as the phyllosphere. As displayed on a heatmap (Figure 5), many taxa were increased on the surfaces after the incubation period with the plant. A *t*-test showed for instance a significant increase for sequences of *Aspergillus ochraceus* (*P* = 0.03), *Agrobacterium* (*P* = 0.03), *Planctomyces* (*P* = 0.01), on surrounding surfaces during plant incubation. *Planctomycetes* were only recently detected since they often belong to the hitherto-uncultured bacteria (Nunes da Rocha et al., 2009). *A. ochraceus* is a soil-borne ascomycetous fungus capable of producing a variety of mycotoxins; however its airborne spores are one of the potential causes of asthma in children and lung diseases in humans.

## Discussion

In the past, humans and pets were identified as important dispersal sources for microbes into the built environment. Single persons can emit up to 10^6^ microbes per person and per hour (Qian et al., 2012; Dunn et al., 2013). We identified an additional effect of house plants, beyond melioration of our mood and indoor air quality, for the quality and quantity of the indoor microbiome. In a proof of principle analysis, we show in this study that plants are an additional important dispersal source in the built environment.

Our study supports our hypothesis that indoor plants contribute substantially to the microbial abundance and diversity in the built environment presented in Berg et al. (2014b) in a pilot experiment. Since plants in general influence abundance and diversity of microbes, they might be important for human wellbeing inside the built environment also from the perspective of plant-associated microbiota. Bacteria and fungi are well-known plant inhabitants, but plant-associated Archaea (*Thaumarchaeota* like *Nitrososphaera* and *Euryarchaeota* like *Halobacteriacae* and *Methanobrevibacter*) have only recently been discovered in olive leaves (Müller et al., 2015). To date, the role of Archaea in the phyllosphere is completely unknown, but their constant occurrence in many common environments might indicate basic functions in many ecosystems (Oxley et al., 2010; Bates et al., 2011; Moissl-Eichinger, 2011; Probst et al., 2013). On average 61% of detected bacterial and fungal sequences were derived from intact cells or spores as revealed by PMA (propidium monoazide) treatment of a subset of samples from all indoor spaces prior to DNA extraction, which masks DNA from compromised cells (Supplementary Table S5). This high rate (relative values) of intact cells from all domains of life might be due to the DNA removal and degradation procedures applied to the chamber prior to plant isolation. This uncommon, and very rigorous procedure might explain a higher proportion of intact cells compared to other indoor environments with strict cleaning procedures such as cleanrooms, with only 1% intact microorganisms, compared to 45% in garment areas (Moissl-Eichinger et al., 2015). Nevertheless, a high proportion of intact cells allow active interactions of microbes in the presence of water and nutrients, which could be tackled by metabolome studies.

The general increase of the microbial population on indoor surfaces was not surprising after such a long time of isolation in an enclosed system, but we were especially interested in identifying differences in diversity as well as sources of the microbial dispersal. Microbial diversity shifted with an increase for bacteria but a decrease for fungi on surrounding wall and floor surfaces as well as plant leaves. This transition could be due to unknown plant properties, but more obvious they might be the result of the altered microclimate inside the chamber after half a year of incubation (Supplementary Figure S1). Hence, the decrease in relative humidity might explain the lowered diversity for fungi on surfaces over time. An increasing microbial diversity on surfaces as well as the higher similarity to plant leaves could be of importance, since microbial diversity was shown to determine the invasion by a bacterial pathogen (Van Elsas et al., 2012). Due to the fact that several microbial indoor pathogens are known to be able to cause severe health problems (Nunes da Rocha et al., 2009), a higher diversity could help to avoid settling of these pathogens. LEfSe and partly the network analysis (Figures 3, 4 and Supplementary Figures S2, S3) revealed the importance of phyllosphere associated microbiota for the transfer of microbes and the general increase of abundance and diversity on the surrounding wall and floor surfaces. This shows that all microenvironments share a part of the microbiome and that house plants act as a bio-resource.

Altogether, plant incubation led to an increase of beneficial plant-associated bacteria *Paenibacillus* (Rybakova et al., 2015), plant-associated *Plantomycetes* with unknown function (Nunes da Rocha et al., 2009) and the spore-producing fungi *Aspergillus ochraceus, Wallemia muriae* and *Penicillium* spp. with allergenic potential (Reponen et al., 2012). The plant microbiome can be altered by the application of biological control agents or stress protection agents (Yang et al., 2009; Berg et al., 2013). This opportunity can be used to develop control agents with beneficial effects to plants as well as to humans. In this context it should also be possible to reduce the proportion of spore-producing fungi, since many of them harbor an allergenic potential (Reponen et al., 2012). Bacterial and fungal biocontrol agents for certain purposes have already been developed (Berg et al., 2013), but the potential of Archaea is completely unknown. Due to the fact that none of the archaeal representatives was judged to be pathogenic so far, they may be a healthy alternative.

Although the plant was identified as major source for microorganisms in a closed cabinet, our experimental design still has several limitations, which will be discussed in detail: Firstly, the study design has some artificial components. The study setup presented here might ignore many other influences between interactions of house plants with their surrounding built environment. However, to limit potential influences and make a compromise of artificial and common environmental parameters, we decided to conduct the experiment in a closed chamber, with ordinary water supply and growing substrate. Secondly, we investigated only one house plant in one incubation system. Due to limitations to reproduce identical indoor environments we focused on one incubation system to limit divergent environmental parameters with unknown effects. As a third point, we only studied two time points. The selection of two sampling points was a compromise to guarantee a low disturbance by the invasive sampling methods. Although more sampling points would help to identify the source of microbial dispersal, we decided against this procedure since regular sampling would disturb microbial abundance and diversity and might increase the level of potential contaminations of the chamber from outside to a critical magnitude.

Additional studies with labeled microorganisms can provide further evidence for microbial dispersal from house plants. House plants are normally grown in soil, which contain a highly diverse microbiome and can influence the environment as well as the phyllosphere as shown by Rastogi et al. (2012).

Indoor plants have the potential to influence the microbiome of the built environment similar to humans and pets. Hence, aside from determining other factors like architecture, ventilation, and room maintenance etc. the microbiome of the built environment is particularly defined by its eukaryotic habitants. The embellishment of built environments with indoor plants does not have an aesthetic relevance alone, indoor plants can act as a simple but efficient way to stabilize and increase diversity of beneficial microbes in the built environment and other enclosed systems for humans in the future such as space stations or manned space missions to successfully colonize other planets.

## Author contributions

AM: study design, performed experiments, analyzed the data, wrote the manuscript; CM: reviewed the study design and manuscript; GB: study design, wrote manuscript.

### Conflict of interest statement

The authors declare that the research was conducted in the absence of any commercial or financial relationships that could be construed as a potential conflict of interest.

## Supplementary material

Supplementary Figure S1 **Microclimate recordings of 1000 measured points of temperature and humidity in the isolation chamber from August to December 2013 (x-axis)**. Black line indicates temperature values in °C and gray line indicates relative humidity recordings in % (y-axis).

Supplementary Figure S2 **Core OTU network of 16S rRNA gene amplicons from plant (green triangles), floor and wall surfaces (squares) and the surrounding indoor air (blue hexagons)**. OTUs (circles) are spring embedded eweighted due to their abundance and distribution (shared OTUs are colored according to their sample origin). Details of network visualizations are given in Moissl-Eichinger et al. (2015).

Supplementary Figure S3 **Core OTU network of ITS region amplicons from plant (green triangles), floor and wall surfaces (squares) and the surrounding indoor air (blue hexagons)**. OTUs (circles) are spring embedded eweighted due to their abundance and distribution (shared OTUs are colored according to their sample origin). Details of network visualizations are given in Moissl-Eichinger et al. (2015).

Supplementary Table S1 **Sequence of primers used in this study**.

Supplementary Table S2 **Summary of obtained raw and quality filtered sequences**.

Supplementary Table S3 **Bacterial OTUs and taxa detected in this study according to sampled indoor spaces (unassigned reads were summarized due to file size limitations)**.

Supplementary Table S4 **Fungal OTUs and taxa detected in this study according to sampled indoor spaces (unassigned reads were summarized due to file size limitations)**.

Supplementary Table S5 **Summary of controls with counts or hits in this study**.
