# Supplementary figures and images for: Microbiome interplay: plants alter microbial abundance and diversity within the built environment
Source: Front Microbiol. 2015 Aug 28;6:887. doi: 10.3389/fmicb.2015.00887 (PMC4552223; doi:10.3389/fmicb.2015.00887)

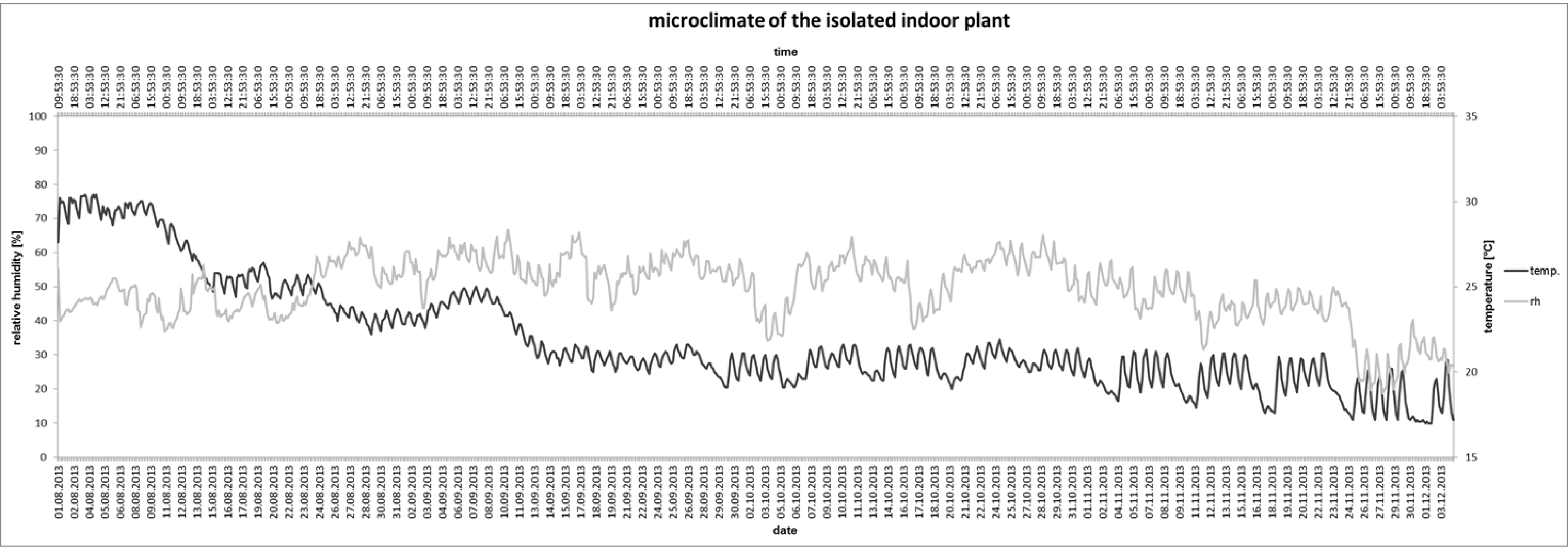

Supplement: Supplementary Figure S1 — Microclimate recordings of 1000 measured points of temperature and humidity in the isolation chamber from August to December 2013 (x-axis). Black line indicates temperature values in °C and gray line indicates relative humidity recordings in % (y-axis). [file Image1.TIF]

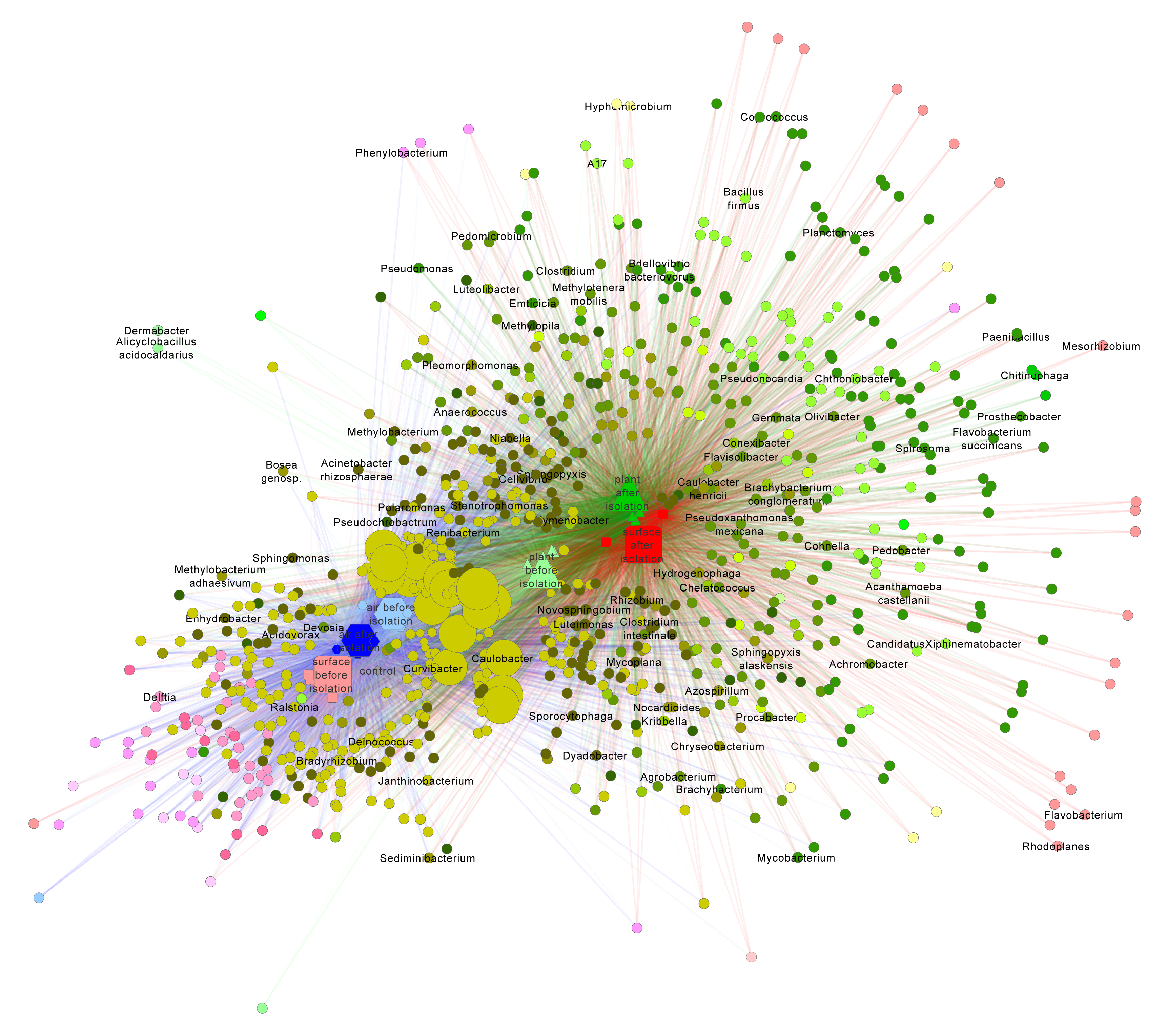

Supplement: Supplementary Figure S2 — Core OTU network of 16S rRNA gene amplicons from plant (green triangles), floor and wall surfaces (squares) and the surrounding indoor air (blue hexagons). OTUs (circles) are spring embedded eweighted due to their abundance and distribution (shared OTUs are colored according to their sample origin). Details of network visualizations are given in Moissl-Eichinger et al. (2015). [file Image2.TIF]

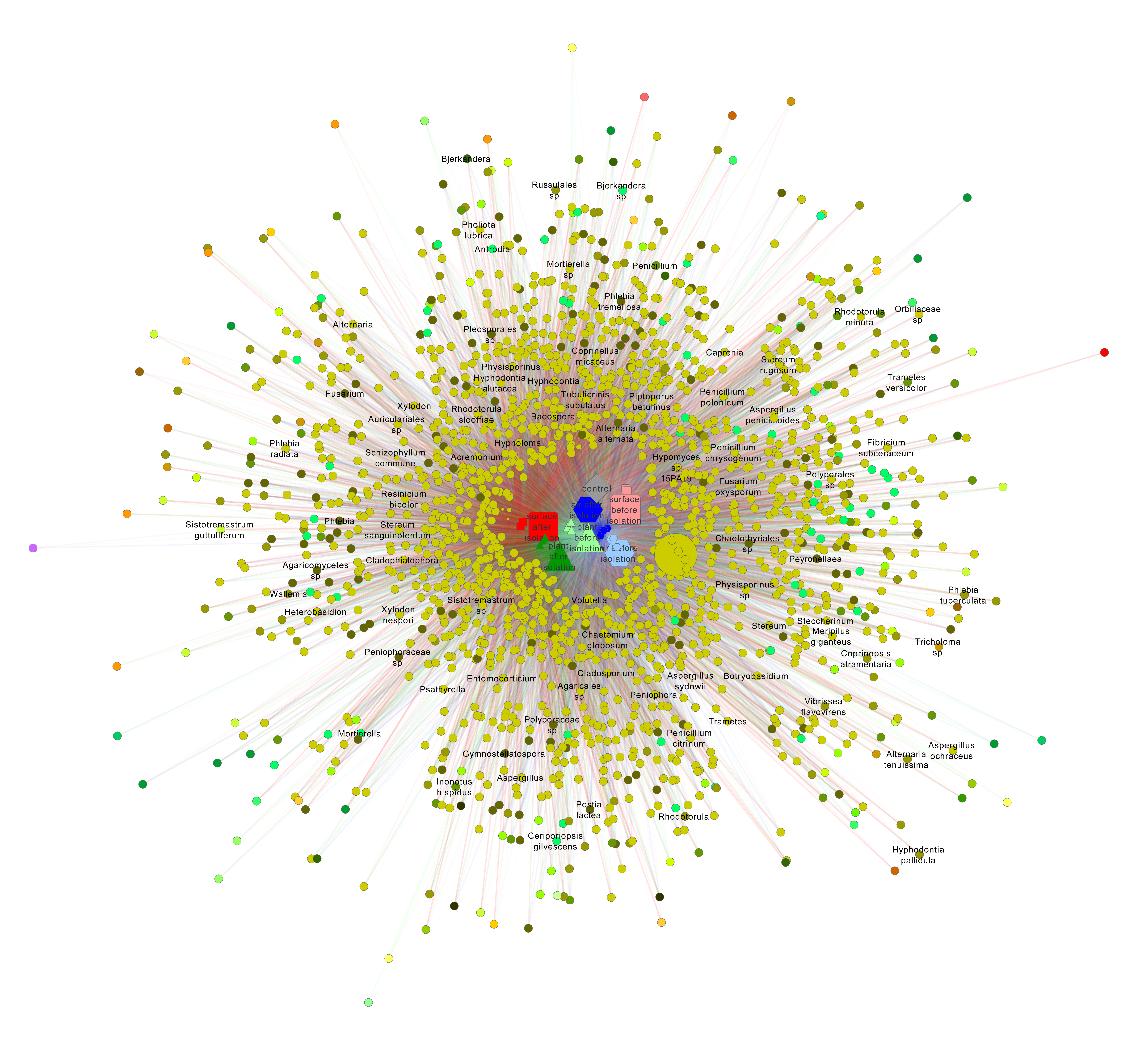

Supplement: Supplementary Figure S3 — Core OTU network of ITS region amplicons from plant (green triangles), floor and wall surfaces (squares) and the surrounding indoor air (blue hexagons). OTUs (circles) are spring embedded eweighted due to their abundance and distribution (shared OTUs are colored according to their sample origin). Details of network visualizations are given in Moissl-Eichinger et al. (2015). [file Image3.TIF]
